# Hepatocyte gene expression and DNA methylation as ancestry-dependent mechanisms in African Americans

**DOI:** 10.1038/s41525-019-0102-y

**Published:** 2019-11-25

**Authors:** C. S. Park, T. De, Y. Xu, Y. Zhong, E. Smithberger, C. Alarcon, E. R. Gamazon, M. A. Perera

**Affiliations:** 1 Department of Pharmacology, Center for Pharmacogenomics, Feinberg School of Medicine, Northwestern University, Chicago, IL USA; 2 Center for Translational Data Science, University of Chicago, Chicago, IL USA; 3 Department of Pathology and Laboratory Medicine, University of North Carolina School of Medicine, Chapel Hill, NC USA; 4 Vanderbilt Genetics Institute and Division of Genetic Medicine, Vanderbilt University School of Medicine, Nashville, TN USA; 5 Data Science Institute, Vanderbilt University, Nashville, TN USA; 6 Clare Hall, University of Cambridge, Cambridge, UK

**Keywords:** Gene expression, Population genetics

## Abstract

African Americans (AAs) are an admixed population with widely varying proportion of West African ancestry (WAA). Here we report the correlation of WAA to gene expression and DNA methylation in AA-derived hepatocytes, a cell type important in disease and drug response. We perform mediation analysis to test whether methylation is a mediator of the effect of ancestry on expression. GTEx samples and a second cohort are used as validation. One hundred and thirty-one genes are associated with WAA (FDR < 0.10), 28 of which replicate and represent 220 GWAS phenotypes. Among PharmGKB pharmacogenes, *VDR*, *PTGIS*, *ALDH1A1*, *CYP2C19*, and *P2RY1* nominally associate with WAA (*p* < 0.05). We find 1037 WAA-associated, differentially methylated regions (FDR < 0.05), with hypomethylated genes enriched in drug-response pathways. In conclusion, WAA contributes to variability in hepatocyte expression and DNA methylation with identified genes previously implicated for diseases disproportionately affecting AAs, including cardiovascular (*PTGIS*, *PLAT*) and renal (*APOL1*) disease, and drug response (*CYP2C19*).

## Introduction

African Americans (AAs) are an admixed population, having varying proportions of African and European ancestry across individuals.^1^ As a consequence of their West African ancestry (WAA), AAs have more genetic variation and shorter extent of linkage disequilibrium than European ancestry populations, with the proportion of WAA varying greatly across self-identified AAs.^2^ The variability in the proportion of admixture in AAs may aid in explaining differences in hepatocyte gene expression and DNA methylation patterns that cannot be elucidated in homogeneous populations such as those of European-only ancestry. For example, WAA has been shown to predict a stronger inflammatory response to pathogens compared to those of European ancestry due to recent selective pressures specific to this population.^3^

Furthermore, AAs suffer disproportionately from many chronic diseases and adverse drug reactions, as compared to other populations,^4,5^ as well as being protected from some conditions. As an example, AAs have a higher risk of cardiovascular events and negative outcomes to therapy such as the antiplatelet drug clopidogrel.^6^ They have higher incidences of death and disability from cardiovascular diseases (CVDs), thrombosis, renal dysfunction and pathologies, diabetes, cancers, and other metabolic disorders.^7–15^ Conversely, they have lower prevalence of disease such as testicular cancer.^16^ Differences in gene expression may help explain these observed differences.

Owing to the key role of the liver in biosynthesis, drug metabolism, and complex human diseases, genetic and epigenetic differences in the liver may be used to uncover the underlying causal genes responsible for chronic diseases that disproportionately affect AAs.^17,18^ Thus comprehensive mapping of liver expression quantitative trait loci (eQTLs) proposed several candidate susceptibility genes associated with type I diabetes, coronary artery disease and plasma cholesterol levels in a white cohort.^19^ More recently, finer-resolution mapping of liver eQTLs, combining both gene expression data with histone modification-based annotation of putative regulatory elements, identified 77 loci found to associate with at least one complex phenotype.^20^ In addition, our group has previously shown that studies specifically investigating the AA-specific genetic variants can reveal population-specific risk factors that may explain differences in drug response, such as African ancestry-specific genetic risk factors associated with a higher risk of bleeding from warfarin therapy in AAs,^12^ as well as population-specific variants associated with increased risk of thrombotic disease.^21^

Rather than identifying disease susceptibilities through genome-wide association studies (GWAS), here we use the association of genomic ancestry to gene expression and DNA methylation to uncover potential drivers of disease and drug response that may explain differences in disease and drug response in AAs. Of the genes we identified, many are known to be dysregulated in diseases that disproportionately affect AAs, and others are associated with drug metabolism.

## Results

### Cohorts

**Fig. 1:**
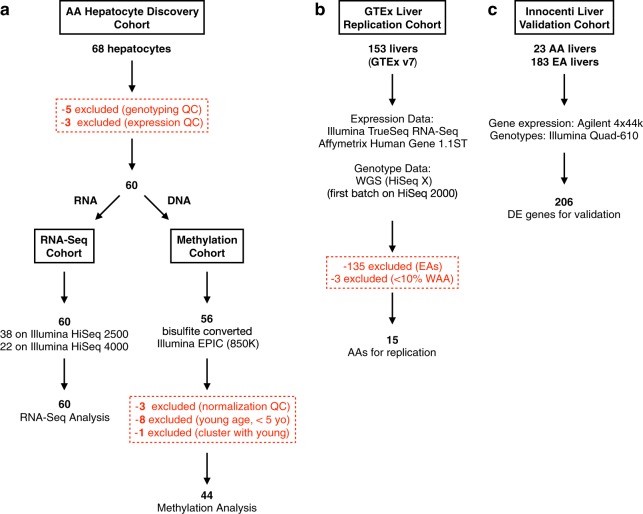
Flowchart outlining each cohort use in the analyses. **a** Hepatocyte Discovery Cohort from AA primary hepatocyte cultures were RNA-sequenced and assessed for DNA methylation. **b** The GTEx Liver Replication Cohort was comprised of AA liver samples with available genotype and gene expression data. **c** Innocenti Liver Validation Cohort was comprised of liver samples from both EAs and AAs obtained through GEO (accession GSE124076), in which differential gene expression was used to validate findings from the Hepatocyte Discovery Cohort

Sixty primary hepatocyte cultures, procured from self-identified AAs, passed all quality control (QC) steps and were used for RNA-sequencing (RNA-seq) analysis (Fig. 1). Fifty-six hepatocyte cultures were assayed for DNA methylation, with 44 used in the final methylation analysis (Fig. 1). We obtained genome-wide genotyping data on 153 subjects from the Genotype-Tissue Expression Project (GTEx) liver cohort, version 7, of which 15 AAs were used as a replication cohort (Fig. 1).^22^ All 60 of our AA hepatocyte cohort were confirmed as having WAA ranging from 41.8% to 93.7%, and 15 subjects in the GTEx liver cohort met the WAA inclusion criteria with WAA ranging from 75.6% to 99.9% (Supplementary Fig. S1). As a quantification of African ancestry, WAA and principal component 1 (PC1) were highly correlated (Supplementary Fig. S2, Pearson correlation = −0.999). The Innocenti Liver Replication Cohort consisting of 23 AAs and 183 European Americans (EAs) was also used to validate our findings from the Hepatocyte Discovery Cohort (Fig. 1). Table 1 shows the demographics of each cohort. The GTEx Replication Cohort and the Innocenti Validation Liver Cohort are comparable to the Hepatocyte Discovery Cohort, but there are differences across groups in age (*p* = 0.0280, specifically between Hepatocyte Discovery Cohort and GTEx Replication Cohort, *p* = 0.0409), as well as sex (*p* = 0.0439) (Table 1). Importantly, there were no differences in proportion of WAA between the cohorts (Table 1).

**Table 1:** Demographics and clinical characteristics of hepatocyte/liver cohorts

	Hepatocyte Discovery Cohort		GTEx Liver Cohort	Innocenti Validation Liver Cohort
	RNA-Seq cohort	Methylation cohort		
Variable	AA (*n* = 60)	AA (*n* = 44)	AA (*n* = 15)	AA^a^ and EA^b^ (*n* = 206)
Percentage of AA subjects (%)	100	100	100	11.1
Age, years^c^ (mean ± SD)	39 ± 20.5	46 ± 12.2	54.5 ± 6.6	46 ± 22
Sex (female %)^d^	48.3	52.3	20	36.4
West African^e^ ancestry (%) (mean ± SD)	78.17 ± 12.86	79.83 ± 11.86	84.04 ± 7.13	NA

^a^AA: African Americans >40% WAA

^b^EA: European Americans with <1% WAA

^c^*p* = 0.0280 for age (between groups, one-way ANOVA), *p* = 0.0409 (between RNA-Seq and GTEx Liver cohorts)

^d^*p* = 0.0439 for sex

^e^*p* = 0.2331 for ancestry

### Genes expressed in hepatocytes and pharmacogenes associated with African ancestry

**Fig. 2:**
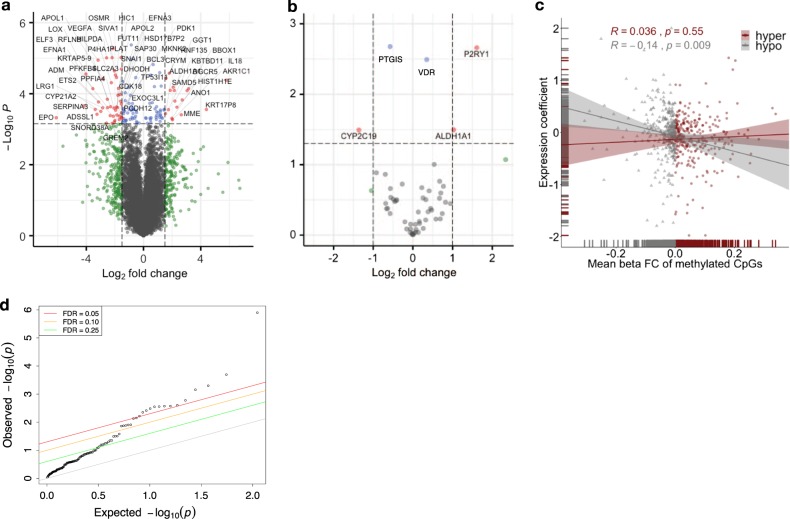
Gene expression traits and methylation patterns associated with West African ancestry in hepatocytes. **a** Enhanced volcano plot of gene expression traits associated with increasing WAA plotted against −log_10_ *p* values of all 18,854 genes expressed in hepatocytes and 131 genes significantly associated with WAA represented as red and blue circles (red circles: FDR < 0.05 and logFC > 1.5 and <−1.5; blue circles: FDR < 0.05 and logFC < 1.5 and >−1.5; green circles: FDR > 0.05 and logFC > 1.5 and <−1.5, gray circles: FDR > 0.05 and logFC < 1.5 and >−1.5) and **b** enhanced volcano plot of gene expression traits associated with increasing WAA plotted against −log_10_ *p* values within the subset of 64 PharmGKB “very important genes” (red circles: *p* < 0.05 and logFC > 1.0; blue circles: *p* value < 0.05 and logFC < 1.0; gray circles: *p* value > 0.05). **c** Correlation of 1034 unique genes containing DMRs significantly associated with WAA (mean Beta fold change from DMRcate) with coefficient of gene expression at each gene (indicating the direction of association to ancestry). Each point represents a gene, with gray triangles representing hypomethylated genes (Pearson’s *r* = −0.014, *p* = 0.009) and maroon red circles representing hypermethylated genes (Pearson’s *r* = 0.036, *p* = 0.55). Gray and maroon hash marks on the *x*- and *y*-axis represent genes plotted with both expression and methylation values. Gray and maroon shading around each regression line re*p*resents the C.I. 95%. **d** Q–Q plot of the observed versus expected −log_10_ *p* values in the replication cohort (*n* = 206). Each point represents a gene with the colored lines representing different FDR thresholds of significance

Association analysis of RNA-seq gene expression traits in the Hepatocyte Discovery Cohort with percentage WAA identified 131 genes, for which gene expression traits were significantly associated with WAA (Supplementary Data S1; Fig. 2a, false discovery rate (FDR) < 0.10). We were able to replicate 28 of these genes in the independent Innocenti Liver Validation Cohort as differentially expressed (DE) between AAs and EAs in the liver^23^ (Table 2; Fig. 2d, *p* < 0.05). These 28 validated genes are associated with 220 reported and mapped disease and measurement traits that have been curated in the NCBI GWAS catalog.^24^ Of these, 118 are unique diseases or traits (Supplementary Data S2).^24^ The phenotypic disease and quantitative GWAS traits associated with WAA include blood and blood pressure measures, coronary heart and artery disease, diabetic blood measures, chronic inflammatory disease, chronic kidney disease, and various cancers.

**Table 2:** Significant and replicated DE genes between AAs and EAs (*p* < 0.05) from a genome-wide discovery of genes associated with West African ancestry (FDR < 0.10)

Gene^b^	AA Hepatocyte Cohort			DE in AA vs EA^a^
	Effect size	*p*	FDR < 0.10	*p* < 0.05^c^
**IL18**	3.152	7.25E−05	0.0471	0.00000126
**APOL1**	−2.848	3.83E−05	0.0380	0.000202
**VEGFA**	−2.246	4.91E−05	0.0421	0.000502
**PARD3**	0.437	1.86E−04	0.0773	0.000696
**SAP30**	−1.945	2.93E−04	0.0788	0.00167
**LRRC37A2**	−0.306	6.73E−04	0.0988	0.00249
**ENO1**	−1.007	1.89E−04	0.0773	0.00266
**PGK1**	−0.929	5.29E−04	0.0882	0.00268
**DGCR5**	2.192	1.62E−04	0.0751	0.00278
**MRO**	1.131	2.57E−05	0.0380	0.00282
**GREM2**	−2.191	6.86E−04	0.0988	0.00327
**CYP21A2**	−2.986	3.17E−04	0.0790	0.00385
**CEBPB**	−1.454	6.36E−04	0.0967	0.00445
**MAD2L1BP**	−0.505	3.04E−04	0.0790	0.00605
**GPR4**	−1.316	3.08E−04	0.0790	0.00706
**RNF149**	−0.833	2.37E−04	0.0788	0.00743
**GPI**	−1.453	4.58E−05	0.0411	0.0123
**SLC22A15**	−1.271	6.86E−04	0.0988	0.0125
**HIC1**	−1.567	9.64E−06	0.0307	0.013
**APOL2**	−1.643	1.06E−04	0.0583	0.0134
**MME**	2.640	3.94E−04	0.0854	0.0137
MKNK2	−1.545	3.44E−04	0.0811	0.0262
MGRN1	−1.010	2.76E−04	0.0788	0.0313
C3orf33	0.506	6.45E−04	0.0973	0.0314
PTPN4	0.484	5.37E−04	0.0882	0.0321
NPR2	1.235	6.11E−05	0.0471	0.0444
MSX1	1.432	2.90E−04	0.0788	0.0448
PDK1	−2.038	6.30E−04	0.0966	0.0498

^a^Replication in differentially expressed (DE) genes between 23 African Americans (AAs) and 183 European Americans (EAs)

^b^Genes with FDR < 0.1 in the Replication Cohort are shown in bold

^c^Replication *p* value (*p* < 0.05) based on 131 discovery genes (Supplemental Data S1)

In the GTEx Liver Replication Cohort of 15 AAs, we were able to replicate 8 of the 131 significant WAA-associated genes: *DHODH* (effect size [confidence interval (C.I.)] = 3.19 [0.03, 6.34], *p* = 0.048), *GPI* (effect size [C.I.] = −3.46 [−6.88, −0.04], *p* = 0.048), *HSD17B7P2* (effect size [C.I.] = −9.22 (−17.0, −1.44], *p* = 0.027), *PLCL2* (effect size [C.I.] = −5.76 [−11.38, −0.15], *p* = 0.046), *SLC2A3* (effect size [C.I.] = −5.95 [−11.17, −0.73], *p* = 0.032), *TRIM39* (effect size [C.I.] = 5.53 [0.75, 10.30], *p* = 0.030), *VEGFA* (effect size [C.I.] = −4.74 [−8.93, −0.56], *p* = 0.032), and *COL26A1* (effect size [C.I.] = −7.13 (−13.67, −0.60], *p* = 0.03), where the effect size represents fold change of the corresponding gene’s expression with increased African ancestry (Supplementary Data S1).

Owing to the importance of the liver in pharmacologic drug response, we conducted a secondary subset analysis to investigate the role of ancestry on gene expression in important pharmacogenes. In this analysis, we used a subset of genes belonging to the very important pharmacogenes (VIP) reported in PharmGKB. These 64 VIP genes are known to be expressed in hepatocytes, and owing to the reduced multiple testing burden, we considered a nominal *p* < 0.05 suggestive of significance. The VIP genes represent drug-metabolizing enzymes, transporters, and drug target genes that are well established for their roles in drug response.^25^ Testing the association between WAA and gene expression in the VIP genes identified five genes that were significantly associated with WAA: *VDR* (effect size = 0.35, *p* = 0.003), *PTGIS* (effect size = −0.57, *p* = 0.002), *ALDH1A1* (effect size = 1.03, *p* = 0.032), *CYP2C19* (effect size = −1.36, *p* = 0.032), and *P2RY1* (effect size = 1.61, *p* = 0.002 (Fig. 2b).

### DNA methylation patterns in hepatocytes are associated with African ancestry

To identify differentially methylated (DM) regions (DMRs) and CpGs associated with WAA, we performed linear regression on each CpG site.^26^ We identified 23,317 significant DM CpG sites, out of a total of 867,531 probes on the Illumina EPIC BeadChip microarray, annotated to 11,151 unique genes (Supplementary Fig. S3a; Benjamini–Hochberg (BH)-adjusted *p* < 0.05). These DM CpGs correspond to 1037 DMRs annotated to 1034 unique genes (Supplementary Fig. S3b; minimum FDR < 0.0001). Each DM CpG site was categorized into hypermethylated (HyprM) and hypomethylated (HypoM) sites: 15,404 HyprM CpG sites constituted 435 HyprM DMRs, mapping to 432 unique genes; 7913 HypoM CpG sites constituted HypoM 602 DMRs, mapping to 602 unique genes; and 7 annotated genes had both HyprM and HypoM DMRs.

As compared to all CpG sites tested, the gene body (45.0% vs 40.9%, *p* < 0.0001, chi-squared test) and shelf (8.4% vs 6.9%, *p* = 0.011, chi-squared test) had significantly higher proportions of DM CpGs, while the promoter (18.4% vs 20.3%, *p* < 0.002, chi-squared test), intergenic regions (IGR) (24.2% vs 27.7%, *p* < 0.0001, chi-squared test), and shore regions (16.3% vs 18.2%, *p* < 0.0022, chi-squared test) had significantly lower proportions of DM CpGs. In our analysis of DM loci associated with WAA, 75.9% of DM CpG sites within islands were HypoM, while the shore, shelf, and open sea were predominantly HyprM (65.5%, 81.7%, and 78.9% respectively; Supplementary Fig. S3c). Within transcriptionally regulated promoter regions, 54.0% of CpG sites were HypoM (Supplementary Fig. S3d).

Within the promoter, 71.5% of DM CpGs were HypoM 200 kb upstream of the transcription start site (TSS), while 42.9% of DM CpGs were HypoM 1500 kb upstream of the TSS (Supplementary Fig. S3d). DNA methylation around TSS is an established predictor of gene expression, with increased methylation leading to decreased expression.^27,28^ In the gene body, 73.0% of DM sites were HyprM (Supplementary Fig. S3d). IGR, 5’-untranslated region (UTR), and 3’-UTR were predominantly HyprM (67.5%, 64.0%, and 79.6% respectively).

Next, we characterized the locations of WAA-associated CpGs by genomic features and gene annotations. Within the 7913 HypoM CpG sites associated with WAA, there was a greater proportion of HypoM CpGs located in islands compared to HyprM CpG sites (15.1% vs 4.8%, *p* < 0.0001, chi-squared test; Supplementary Fig. S3c). Within the 15,404 HyprM CpGs associated with WAA, there was a greater proportion of HyprM CpGs compared to HypoM CpGs in the open sea regions (43.8% vs 11.7%, *p* < 0.0001, chi-squared test), shelf (6.9% vs 1.5%, *p* < 0.0001, chi-squared test), and shore (10.6% vs 5.6%, *p* < 0.0001, chi-squared test) (Supplementary Fig. S3c). By genomic annotation, the promoter had greater proportion of HypoM CpG sites (46.0% vs 54.0%, *p* < 0.0001, chi-squared test), while the gene body (72.9% vs 27.1%, *p* < 0.0001, chi-squared test), IGR (67.5 vs 32.5%, *p* < 0.0001, chi-squared test), 5’-UTR (63.9% vs 36.1%, *p* < 0.0001, chi-squared test), and 3’-UTR (79.5% vs 20.5%, *p* < 0.0001, chi-squared test) had greater proportions of HyprM CpG sites (Supplementary Fig. S3d).

### Gene expression trait correlation to WAA-associated DNA methylation patterns

To determine the relationship of WAA-associated DMRs with gene expression traits, we looked at the association of the 1034 unique genes corresponding to the 1037 DMRs with their respective gene expression profiles. Although there was no correlation of WAA-associated HyprM gene regions with gene expression (Fig. 2c, Pearson’s *r* = 0.036, *p* = 0.55), HypoM gene regions associated with WAA were negatively correlated with gene expression (Fig. 2c, Pearson’s *r* = −0.14, *p* = 0.009). In general, all genes within DMRs associated with WAA also showed negative correlation between gene expression and methylation (Pearson’s *r* = −0.1, *p* = 0.013).

**Fig. 3:**
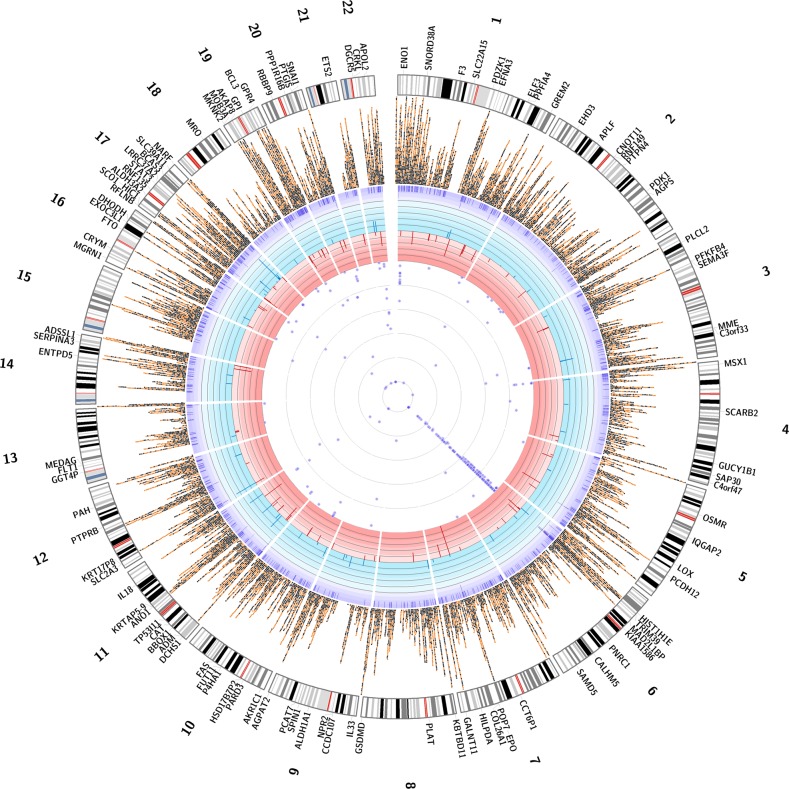
A Circos plot of significantly associated CpGs, DMRs, and gene expression traits associated with West African ancestry and GWAS catalog traits associated with replicated gene expression traits. The innermost ring represents the 220 GWAS catalog traits associated with the 28 genes replicated in the Innocenti et al. dataset (*p* < 0.05) with each purple circle representing the scaled −log(*p* value) of a study from the GWAS catalog. The second ring represents 83 genes associated with decreased expression with increased WAA (red bars represent fold change, ranging from 0 to −6). The third ring represents 48 genes associated with increased expression with increased WAA (blue bars represent fold change >1.5, ranging from 0 to +6). The fourth ring represents 1037 DMRs significantly associated with WAA (purple tiles represent DMRs that stack when overlapping). The fifth ring represents 23,317 significant differentially methylated CpGs (black squares represent 7913 hypomethylated CpG sites; orange circles represent 15,404 hypermethylated CpG sites; not all CpGs are depicted due to reduced crowding implemented in the Circos program). The next ring represents the karyotype of the human genome (reference hg38) and the outermost ring corresponds to the gene names of the 131 WAA-associated gene expression traits identified

From the 131 gene expression traits significantly associated with WAA, we identified an overlap of ten DM gene regions (Supplementary Data S3a). Of these ten, five genes (*COL26A1*, *HIC1*, *MKNK2*, *RNF135*, and *TRIM39)* showed concordant directions of effect (e.g., increased methylation leading to decreased gene expression). To determine whether methylation mediated the association of WAA to gene expression in these five genes, we performed mediation analysis. We found that the expression of two of these genes was mediated by methylation: *COL26A1* (mediated effect = 2.336, 95% C.I. = 0.563–4.39, *p* < 2E−16) and *MKNK2* (mediated effect = 1.095, 95% C.I. = 0.016–2.61, *p* = 0.046) (Supplementary Data S3b and S3c). A comprehensive Circos plot summarizes these findings: 23,317 HypoM and HyprM CpG sites, 1037 DMRs, 131 genes significantly associated with WAA, and 220 GWAS disease traits from the GWAS Catalog that are associated with the 28 validated genes (Fig. 3, Supplementary Data S1).

### Functional representation of WAA-associated genes and DM genes associated with African ancestry

**Fig. 4:**
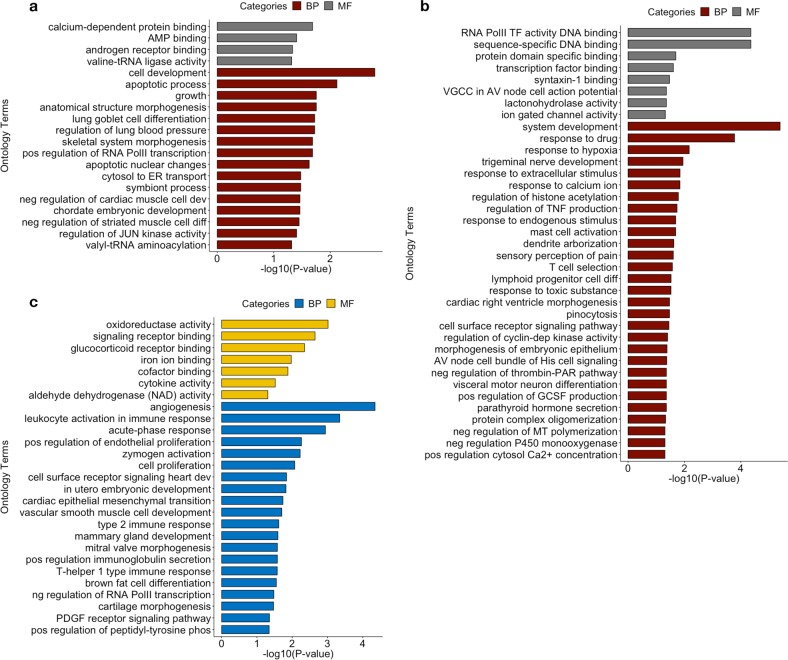
Enrichment of biological processes and molecular functions of differentially methylated genes associated with West African ancestry and corresponding gene expression traits. **a** Gene ontology terms that are enriched for biological processes (BP) and molecular functions (MF) for 432 genes annotated to differentially hypermethylated regions, **b** 602 genes annotated to differentially hypomethylated regions, and **c** 131 genes with gene expression traits associated with WAA (FDR < 0.10). *p* Values are BH-adjusted *p* values obtained from gProfiler

To understand the biological relevance of differentially HyprM and HypoM genes associated with WAA, we performed Gene Ontology (GO) analysis of the 432 unique genes comprised of the 435 HyprM DMRs and 602 unique genes comprised of the 602 HypoM DMRs using a gene panel of all annotated genes in the GO database. HyprM genes are enriched for “cell development” (BH-adjusted *p* = 0.0016) and “apoptotic process” (BH-adjusted *p* = 0.0076) within the ontology of biological processes (Fig. 4a). HypoM genes were enriched for “system development” (BH-adjusted *p* = 4.0 × 10^−6^), “response to drug” (BH-adjusted *p* = 1.7 × 10^−4^), and “response to hypoxia” (BH-adjusted *p* = 0.0068) within the ontology of biological processes (Fig. 4b). In addition, HypoM genes are enriched for “sequence-specific DNA binding” (BH-adjusted *p* = 4.4 × 10^−5^) and “RNA polymerase II transcription factor activity” (BH-adjusted *p* = 4.4 × 10^−5^) within the ontology of molecular functions (Fig. 4b).

With respect to GO analysis of the 131 WAA-associated gene expression traits in hepatocytes (FDR < 0.10), “angiogenesis” (BH-adjusted *p* = 4.5 × 10^−5^), “leukocyte activation involved in immune response” (BH-adjusted *p* = 4.5 × 10^−4^), “acute-phase response” (BH-adjusted *p* = 0.0011), “positive regulation of endothelial cell proliferation” (BH-adjusted *p* = 0.0055), “zymogen activation” (BH-adjusted *p* = 0.0059), and “cell proliferation” (BH-adjusted *p* = 0.0085) were biological processes that showed significant enrichment (Fig. 4c). Molecular functions, including “oxidoreductase activity” (BH-adjusted *p* = 9.6 × 10^−4^), “signaling receptor binding” (BH-adjusted *p* = 0.0023), “glucocorticoid receptor binding” (BH-adjusted *p* = 0.0045), and the KEGG pathway “HIF-1 signaling pathway” (BH-adjusted *p* = 8.2 × 10^−4^), were also enriched (Fig. 4c).

## Discussion

Several studies have shown that the first several PCs of methylation data can capture population structure in cohorts composed of European and African ancestry individuals.^29^ Recently, it was shown that genetic ancestry can be used as a proxy for uncovering unknown covariates contributing to epistatic and gene–environment interactions in both gene expression and DNA methylation data.^30^ A recent study reported that approximately 75% of variation in DNA methylation was attributable to shared genomic ancestry.^31^ This variability in epigenetic processes, accounted for by ancestry, however, may include a genetic component, demographic history (e.g., Transatlantic migration), environmental exposures, and a disease-altered component. Moreover, clinically meaningful measures can be associated with the proportion of WAA, as has been shown for lung function prediction.^32^

In our study, we investigated population-specific gene expression and DNA methylation in AAs, an admixed population. We found HypoM genes, which indicate increased gene expression, are enriched for “system development” and “response to drug.” HypoM, or demethylation, may be a better predictor of gene expression than methylation, which, in contrast to demethylation, may or may not affect gene expression depending on the gene region methylated or the pattern of methylation. For example, methylation within the TSS of the promoter is well known to repress gene expression while methylation within the gene body results in more variable expression.^28,33^

Among gene expression traits associated with WAA, we identified enrichment for genes within the angiogenesis pathway, as well as inflammatory response categories including “leukocyte activation in immune response,” “acute-phase response,” “positive regulation of vascular endothelial proliferation,” “zymogen activation,” and “cell proliferation.” Angiogenesis and inflammatory response pathways may underlie conditions in which AAs may be more susceptible, such as CVD and other chronic inflammatory disease. In particular, *APOL1*, *PTGIS*, and *PLAT* expression levels, which we show to be associated with WAA, have been shown to increase CVD risk and renal disease in AAs.^13,34,35^ Other genes associated with WAA include *ALDH1A1*, which is involved in alcohol and aldehyde metabolism disorders and cancer risk^11,36,37^; *IL-33*, which is involved in beneficial immune response,^38–40^ and *VEGFA*, which has been linked to renal disease and microvascular complications of diabetes.^7,14,41,42^ These findings will require further validation to better elucidate the extent to which the identified pathways affect diseases in AAs.

Of the 131 genes associated with WAA, we were able to replicate a quarter within our GTEx Liver Replication Cohort and the Innocenti Liver Validation Cohort (Table 2). We also identified an overlap with five significantly DM genes with concordant directions of effect, *COL26A1*, *HIC1*, *MKNK2*, *RNF135*, and *TRIM39*. *HIC1* is a potential tumor suppressor that has been linked to poorer outcomes in laryngeal cancer in AAs.^43,44^ *RNF135* is a ring finger protein that is regulated by several population-specific variants.^45^ RNF135, itself, then regulates other genes at distant loci and has been implicated in glioblastomas and autism.^45–47^ Of particular interest for African ancestry populations, *RNF135* has been found to be under selective pressure, specifically in African populations.^48,49^

Among the PharmGKB VIP genes, we identified five genes associated with WAA: *ALDH1A1*, *CYP2C19*, *P2RY1*, *PTGIS*, and *VDR*.^22,25^ Of particular importance, we found that, for every 1% increase in African ancestry, there was a corresponding 1.36% decrease in *CYP2C19* expression and 1.61% increase in *P2RY1* expression. *CYP2C19* is involved in the metabolism of many commonly prescribed drugs, including clopidogrel, an antiplatelet therapy widely used for thrombo-prophylaxis of CVDs and linked to substantial inter-patient differences in drug response. It is also known to inhibit the *P2RY* family of receptor proteins on the surface of platelets.^34,35,50,51^ By inhibiting *P2RY12* function, clopidogrel indirectly suppresses platelet clustering and clot formation and prevents clots contributing to heart attack, stroke, and deep vein thrombosis.^52–54^ *P2RY1* works in concert with *P2RY12* to promote platelet activation and aggregation.^55^ Consequently, *P2YR1* variants have been associated with increased platelet response to adenosine 5’-diphosphate stimulation^56^ and increased expression may be linked to thrombotic disease.^57^

Clopidogrel requires *CYP2C19*-mediated conversion to its active form, but it has been shown that different populations have different levels of CYP2C19 activity.^12,58–61^ The underlying mechanism is multifactorial and genetic polymorphisms contribute to variable drug response within an individual and across populations.^6,62,63^ A study conducted across 24 U.S. hospitals showed 1-year mortality rates of 7.2% in clopidogrel-treated AAs, compared to 3.6% for Caucasians on clopidogrel.^6^ This study also found that AAs were at a higher risk of cardiovascular events and mortality from poor antiplatelet response to clopidogrel. Our finding that *CYP2C19* expression is reduced with WAA while *P2RY1* expression is inversely increased suggests that clopidogrel resistance and susceptibility of AAs to thrombotic disease may be due to ancestry-associated gene expression as an underlying mechanism.

Another WAA-associated gene was *PLAT*, for which gene expression is decreased with increased WAA. The *PLAT* gene, which is involved in plasminogen activation and encodes tissue plasminogen activator (t-PA), is linked to thrombosis and increased risk of CVD.^64,65^ In AAs, polymorphisms in *PLAT* have been linked to CVD and higher levels of t-PA antigen have been observed in both myocardial infarction and venous thromboembolism.^66^ In addition, increased plasma fibrinogen level, which is involved in the fibrinolytic pathway and regulated by t-PA, has been linked to increased venous thrombosis risk in AAs.^67,68^

*VDR* is also important in health and disease because Vitamin D and its active metabolite 1,25(OH)_2_D are exogenous hormones created by sun exposure or through diet, with higher risk for deficiencies in both Vitamin D and its bioactive metabolites in AAs.^69,70^ Those of African ancestry are known to have lower plasma 1,25(OH)_2_D levels. One potential mechanism may be the upregulation of *VDR* with increased WAA to compensate for these lower levels. However, focused studies would need to confirm this hypothesis. In addition, *VDR* single-nucleotide polymorphisms (SNPs) have been implicated in CVDs and various cancer susceptibilities in AAs.^8,9,15,71,72^ SNPs within *VDR* may contribute to an already deficient Vitamin D environment in AAs.

Several limitations exist in this study. First, we were limited by the 60 primary hepatocyte cultures used in this analysis, which reduced our power to detect small changes in gene expression associated with ancestry. Second, the GTEx replication liver cohort, with 15 AA livers, also lacked power to replicate our findings (although we supplement this with a much larger Innocenti Validation Liver dataset for differential expression analysis between AAs and EAs). Third, while most of the genes found in the PharmGKB VIP subset analysis were not statistically significant in the comprehensive genome-wide analysis, we found *ALDH1A1* and *PTGIS* to be significantly associated with WAA in both the genome-wide and in the 64 PharmGKB VIP analysis, and we replicated 8 of the 131 significant WAA-associated genes in a GTEx liver cohort of 15 AAs. More importantly, and the primary reason for the low power in our dataset, or any other dataset available for AAs, very few genome-wide datasets of both genotype and gene expression exist for AAs, and those that do exist have similarly under-powered cohort sizes.

Another differentiation, though not considered a limitation, was that we used cultured hepatocytes as opposed to frozen liver tissues, as was the case with GTEx. Primary cultures may show differences in gene expression profiles from those seen in the intact organ, which consist of approximately 60% hepatocytes.^73^ However, our study design has the advantage of assaying only the gene expression of a single-cell type as opposed to the multiple-cell types found in the liver. Previous studies have shown that primary human hepatocytes show similar gene expression levels for both Phase I (CYPs) and Phase II (e.g., UGTs) drug-metabolizing enzymes to those obtained from frozen liver tissue.^74,75^ Also, the use of primary hepatocyte cultures reduces the effect of environmental confounders inherent in liver tissue (i.e., there is less effect of previous drug/disease exposure) due to controlled tissue culture processes following hepatocyte isolation. The artifact of previous drug/disease exposures is typically present in all transcriptome studies conducted in postmortem human liver tissue. However, as with other studies, we too derived our cultures from non-transplantable livers, which may bias our study to less healthy individuals. Because of the anonymous nature of these samples, we also lack clinical and environmental factors that may affect gene expression.

Currently, genome-wide genetic, epigenetic, and multi-omics datasets of AAs are generally lacking in both the scientific literature and in public databases and repositories. Since genomics studies should be inclusive of all populations to comprehensively unravel disease etiology, the dearth of genetic data in diverse and underrepresented populations poses a major scientific and medical dilemma in new drug development, precision medicine, and public health policies. An archetypal example is the rs12777823 SNP in *CYP2C9*, which was found to associate with lower warfarin dose requirement, but only in AAs.^76^ Findings, such as these, were only made possible due to focused studies of an underrepresented minority patient population.

In conclusion, our study has important implications in the use of genetic ancestry in understanding phenotypic differences and health disparities in AAs. Our study also has major implications for future investigations of genetic factors and potential for drug-response outcomes in admixed populations. As evidenced by the limited genome-wide data in AAs in public databases and biobanks, our study further illustrates the need for inclusion of diverse populations.

## Methods

### Cohorts

A total of 68 AA primary hepatocyte cultures were used for this study. Cells were either purchased from commercial companies (BioIVT, TRL, Life technologies, Corning, and Xenotech) or isolated from cadaveric livers using a modified two-step collagenase perfusion procedure.^77^ Liver specimens were obtained through collaborations with Gift of Hope, which supplies non-transplantable organs to researchers. In addition, we used GWAS data for 153 subjects from GTEx release version 7; of the 153 subjects, 15 were identified as AA and used as a replication set. The Institutional Review Board of Northwestern University has waived the need for approval as this study used human samples obtained from deceased individuals and was thus not considered human subjects’ research.

### Primary hepatocyte isolation

Cadaveric livers obtained from Gift of Hope were transferred to the perfusion vessel Büchner funnel (Carl Roth) and the edge was carefully examined to locate the various vein and artery entries that were used for perfusion. Curved irrigation cannulae with olive tips (Kent Scientific) were inserted into the larger blood vessels on the cut surface of the liver piece. The liver was washed by perfusion of 1 L Solution 1 (HEPES buffer, Sigma-Aldrich), flow rate 100–400 mL/min, with no recirculation, followed by perfusion with 1 L of Solution 2 (EGTA buffer, Sigma-Aldrich), flow rate 100–400 mL/min, with no recirculation. The tissue was washed to remove the EGTA compound by perfusion of 1 L Solution 1, flow rate 100–400 mL/min, with no recirculation. The liver was digested by perfusion with Solution 3 (collagenase buffer, Sigma-Aldrich), flow rate 100–400 mL/min, with recirculation. Following perfusion, liver section was placed in a crystallizing dish (Omnilab) containing 100–200 mL of Solution 4 (Bovine Serum Albumin, Sigma-Aldrich). The Glisson’s capsule was carefully removed and the tissue was gently shaken to release hepatocytes. The cell suspension was then filtered by a 70-μm nylon mesh (Fisher Scientific), and centrifuged at 72 × *g* for 5 min at 4 °C. The pellets contained hepatocytes that were washed twice with Solution 4 and resuspended in plating medium (Fisher Scientific).

For primary hepatocyte cultures, cell viability was determined by trypan blue (Lonza) exclusion using a hemocytometer (Fisher Scientific).^78^ If viability was low, Percoll gradient (Sigma-Aldrich) centrifugation of cell suspensions was carried out to improve the yield of viable cells. Cell were plated at a density of 0.6 × 10^6^ cells/well in 500 µL InVitroGro-CP media (BioIVT, Baltimore, MD) in collagen-coated plates with matrigel (Corning, Bedford, MA) overlay and incubated overnight at 37 °C. Cultures were maintained in InVitroGro-HI media (BioIVT) supplemented with Torpedo antibiotic mix (BioIVT) per the manufacturer’s instructions. The media was replaced every 24 h for 3 days. RNA was extracted after 3 days using the RNAeasy Plus Mini-Kit (Qiagen) per the manufacturer’s instructions.

### Genotyping and QC

DNA was extracted from 1–2 million cells from each hepatocyte line using the Gentra Puregene Blood Kit (Qiagen) as per the manufacturer’s instructions. All DNA samples were then bar-coded for genotyping. SNP genotyping was conducted on the Illumina Multi-Ethnic Genotyping array (MEGA) at the University of Chicago’s Functional Genomics Core using standard protocols.

QC steps were applied, as previously described, with imputation info metric threshold of >0.4.^21^ Briefly, a sex check was performed on genotypes using PLINK (version 1.9) to identify individuals with discordant sex information. Duplicated or related individuals were identified using identity-by-descent method with a cutoff score of 0.125, which indicates third-degree relatedness. A total of five individuals were excluded after genotyping QC analysis. SNPs located on the X and Y chromosomes and mitochondrial SNPs were excluded. SNPs with a missing rate of >5% or those that failed Hardy–Weinberg equilibrium tests (*p* < .00001) were also excluded.

### African ancestry measurement

The genotypes of 68 primary hepatocytes and 153 GTEx subjects were merged with HapMap phase 3 reference data from four global populations: Yoruba in Ibadan, Nigeria (YRI); Utah residents with Northern and Western European ancestry (CEU); Han Chinese in Beijing, China (CHB); and Japanese in Tokyo, Japan (JPT).^79^ Population structure of the merged data was inferred by the Bayesian clustering algorithm STRUCTURE deployed within fastStructure v1.0 and performed without any prior population assignment. We employed the admixture model, and the burn-in-period and number of Markov Chain Monte Carlo repetitions were set to 20,000 and 100,000, respectively.^80^ The number of parental populations (*K*) was set to 3, purporting three main continental groups (African, European, or Asian). WAA percentages of the primary hepatocytes and GTEx subjects were calculated as the probability of being grouped as Yoruba African, Caucasian, and East Asian, respectively.^80^ All individuals in our 60 AA cohort had WAA >40%.

### GTEx replication liver cohort

Of the 153 GTEx liver IDs extracted from “GTEX_Sample_Attributes” file, five were replicates and therefore removed from the analysis. Among the remaining 139 unique liver IDs, 118 had available genotype information for ancestry determination. Individuals with WAA >40% were included in the GTEx AA Liver cohort. After WAA estimation, normalized gene expression reads for 15 subjects meeting the ancestry inclusion criteria were extracted from GTEx expression file (GTEx_Liver.v7.normalized_expression.bed). Age and sex information of these subjects were extracted from the subject phenotype file on the GTEx Portal site (GTEx_v7_Annotations_SubjectPhenotypesDS.txt). Genes were deemed replicated if they showed both significant association with WAA (*p* < 0.05) and concordant direction of effect.

### Validation analysis in an independent liver transcriptome dataset

Gene expression and genotype information for the Innocenti Liver Validation Cohort was obtained from the GEO database (Accession: GSE124076). The gene expression profiling in the liver had previously been conducted in 206 samples using Agilent-014850 4 × 44 *k* arrays (GPL4133).^23,81^ These samples had come from donor livers not intended for organ transplantation. Genotyping on these samples had been done using the Illumina Human 610 quad beadchip platform (GPL8887) at the Northwestern University Center for Genetic Medicine Genomics Core Facility and imputation was subsequently performed using BIMBAM.^82^ Principal component analysis (PCA) was used to quantify ancestry using the Human Genome Diversity Panel with African and European populations as reference, as previously described.^23^

We conducted differential expression analysis between the European samples and the AA samples using Linear Models for Microarray Data (*limma*) as implemented in the Bioconductor package.^83^ This Bayesian methodology uses a “moderated” *t*-statistic from the posterior variance assuming an inverse chi-squared prior for the unknown variance for a gene. We used Bonferroni-adjusted *p* < 0.05 based on the total number of genes that were tested for replication. Genes were deemed validated if they were significantly DE between populations (*p* < 0.05) with a concordant direction of effect (i.e., increased expression in AAs and increased expression with higher WAA).

### RNA-seq and QC

Total RNA was extracted from each primary hepatocyte culture after 3 days in culture using the Qiagen Rneasy Plus Mini-Kit per the manufacturer’s instructions. RNA-QC was performed using an Agilent Bio-analyzer and samples with RNA integrity number scores >8 were used in subsequent sequencing. RNA-seq libraries were prepared for sequencing using the Illumina mRNA TruSeq RNA Sample Prep Kit, Set A (Illumina catalog # FC-122-1001) according to the manufacturer’s instructions. The cDNA libraries of 38 and 22 samples were prepared and sequenced using Illumina HiSeq 2500 and HiSeq 4000 machines, respectively, by the University of Chicago’s Functional Genomics Core to produce single-end 50 bp reads with approximately 50 million reads per sample. Batch effects were corrected for in QC below.

Quality of the raw reads was assessed by FastQC (version 0.11.2). Fastq files with a per base sequence quality score >20 across all bases were included in downstream analysis. Reads were aligned to human Genome sequence GRCh38 and comprehensive gene annotation (GENCODE version 25) was performed using STAR 2.5. Only uniquely mapped reads were retained and indexed by SAMTools (version 1.2). Nucleotide composition bias, GC content distribution, and coverage skewness of the mapped reads were further assessed using read_NVC.py, read_GC.py, and geneBody_coverage.py scripts, respectively, from RSeQC (version 2.6.4). Samples without nucleotide composition bias or coverage skewness and with normally distributed GC content were reserved. Lastly, Picard CollectRnaSeqMetrics (version 2.1.1) was applied to evaluate the distribution of bases within transcripts. Fractions of nucleotides within specific genomic regions were measured for QC and samples with >80% of bases aligned to exons and UTRs were considered for subsequent analysis.

### RNA-seq data analysis

Post alignment and QC, reads were mapped to genes referenced with comprehensive gene annotation (GENCODE version 25) by HTSeq (version 0.6.1p1) with union mode and minaqual = 20.^84^ HTSeq raw counts were supplied for gene expression analysis using Bioconductor package DESeq2 (version1.20.0).^85^ Counts were normalized using regularized log transformation and PCA was performed in DESeq2. PC1 and PC2 were plotted to visualize samples’ expression pattern. Three samples with distinct expression patterns were excluded as outliers resulting in 60 samples used in RNA-seq analysis. We calculated TPM (transcript per million) by first normalizing the counts by gene length and then normalizing by read depth.^86^ Gene expression values were filtered based on the expression thresholds >0.1 TPM in at least 20% of samples and ≥6 reads in at least 20% of samples as performed by the GTEx consortium (https://gtexportal.org, Analysis Methods, V7, updated 09/05/2017).

WAA percentage, gender, age, platform, and batch were used as covariates for downstream analysis. Probabilistic estimation of expression residuals (PEER) method v1.3 was used to identify PEER factors. Linear regression was run on inverse normal transformed expression data with five PEER factors as covariates, on the basis of GTEx’s determination of the number of factors as a function of sample size.^87,88^ WAA-associated genes were identified from a genome-wide list of 18,854 genes for our hepatocyte-derived AA cohort samples and replicated in 21,730 genes from the GTEx-derived AA cohort at FDR cutoff of 0.10. The top 131 genes with an FDR < 0.10 were also replicated in the independent Replication Cohort obtained from Innocenti et al. (GEO Accession number GSE124076).^23^ To investigate whether our replicated gene set may inform previous GWAS association findings, we downloaded NHGRI-EBI GWAS Catalog file (v.1.0.2, 2019-03-22) and kept associations that passed the genome-wide significant level (*p* < 5E−8). The 28 replicated genes were then overlapped with the reported and mapped genes in this file to identify GWAS-associated SNPs linked to our replicated genes.^24^ Unique traits were identified by further filtering the Disease/Trait category from this file to identify unique phenotypes that overlapped with the replicated genes. FDR calculations from linear regressions on gene expression were conducted with the “p.adjust” function in R and the default method of “fdr” was used.

In addition, we also conducted a subset analysis with 64 PharmGKB “VIPs.” These genes have extensive literature support for association with drug responses. We used a nominal *p* value cutoff of 0.05 due to the smaller number of genes being tested.

### Methylation sample preparation and data analysis

DNA was isolated from hepatocytes or liver tissue. Liver tissue was homogenized in a bead mill (Fisher Scientific) using 2.8 mm ceramic beads. Then DNA from hepatocytes or liver was extracted with the Gentra Puregene Blood Kit (Qiagen) as per the manufacturer’s instructions. One microgram of DNA was bisulfite converted at the University of Chicago Functional Genomics Core using standard protocols. CT-conversion was performed using Zymo-Research EZ DNA Methylation Kits and further processed for array hybridization using Illumina provided array reagents. Following hybridization, the arrays were stained per the manufacturer’s protocol and analyzed using an Illumina HiSCAN. Of the 60 available hepatocyte cultures, only 56 produced sufficient bisulfite-converted DNA for analysis.

Illumina MethylationEPIC BeadChip microarray (San Diego, Ca, USA), consisting of approximately 850,000 probes, predefined and annotated,^89^ and containing 90% of CpGs on the HumanMethylation450 chip and with >350,000 CpGs regions identified as potential enhancers by FANTOM5^90^ and the ENCODE project,^91^ was used for methylation profiling of DNA extracted from 56 AA hepatocytes that overlapped the samples used for gene expression analysis. Raw probe data were analyzed using the ChAMP R package for loading and base workflow,^26^ which included the following R packages: BMIQ for type-2 probe correction method;^92^ ComBat for correction of multiple batch effects including Sentrix ID, gender, age, slide, and array;^93,94^ svd for singular value decomposition analysis after correction;^95^ limma to perform differential methylation analysis on each CpG site with WAA as a numeric, continuous variable;^96^ DMRcate for identification of DMRs, using default parameters, and the corresponding number of CpGs, minimum FDR (minFDR), Stouffer scores, and maximum and mean Beta fold change values;^97^ minfi for loading and normalization;^98^ missMethyl for gometh function for GSEA analysis;^99^ and FEM for detecting DM gene modules.^28^

Methylation data QC in ChAMP’s *champ.load()* and *champ.filter()* function removed the following probes: 9204 probes for any sample that did not have a detection *p* value <0.01 and thus considered as a failed probe, 1043 probes with a bead count <3 in at least 5% of samples, 2975 probes with no CG start sites, 78,753 probes containing SNPs,^100^ 49 probes that align to multiple locations as identified by Nordlund et al.,^101^ and 17,235 probes located on X and Y chromosomes. Threshold for significantly DM probes was set at BH-adjusted *p* value of 0.05 for multiple testing, as implemented in the *limma* package. Significance for DMRs was set at adjusted FDR < 0.05 in the DMRcate package using default parameters. Analysis was performed with the R statistical software (version 3.4.3 and version 3.5 for ChAMP (version 2.10.1)). Three outliers, eight samples from young subjects aged <5 years, and one subject who had a similar profile as young and clustered with the eight young subjects, were excluded owing to known differences in methylation profiles associated with age,^102^ leaving 44 samples in the analysis (Fig. 1).

GO analysis was performed with g:Profiler (biit.cs.ut.ee/gprofiler) using the g:GOSt package to provide statistical enrichment analysis of our significantly HypoM and HyprM genes and significantly expressed genes associated with African ancestry.^103^ We filtered for significance at a BH-adjusted *p* value < 0.05. We set hierarchical sorting by dependencies between terms on the strongest setting, “best per parent group,” and only annotated genes were used as the statistical domain size parameter to determine hypergeometrical probability of enrichment. We considered ontology terms to be statistically significant at a BH-adjusted *p* value <0.01.

Correlation of log fold change in DNA methylation of DM CpG sites by genomic feature was performed on significantly DM probes identified by limma and plotted using ggplot2 v3.1.0. Genomic features and transcriptionally regulated regions were subdivided for terms “island,” “open sea,” “shelf,” “shore” and “5’UTR,” “TSS 1500,” “TSS 200,” “Body,” “3’UTR,” respectively, where “island” refers to 1 kb regions of high CpG density, “shore” refers to 2 kb regions flanking “islands,” “shelf” refers to 2 kb regions flanking “shores,” and “open sea” refers to IGRs.^104,105^ Location of CpGs in relation to gene annotation was defined as within the 5’-UTRs, within 1500 bp of the TSS (TSS 1500), within 200 bp of the TSS (TSS 200), within the gene body (Body), or within the 3’-UTR. A chi-squared test was used for all categorical comparisons. Correlation with methylation and gene expression was performed using the ggscatter function in ggpubr v0.2, with the correlation method set to Pearson, C.I. set at 95%, and regression calculations included for the following subsets: HypoM, HyprM, and cumulative CpG sites. Gene name conversions and annotated gene attributes were determined by BioMart v2.38.0. The Circos plot was created using the Circos tool v0.69-6.

Mediation analysis was performed using the R package “mediation” v4.4.7. We first performed linear regression to test whether African ancestry affects methylation for each of the five genes we discovered to be associated with WAA. For each non-zero effect, we subsequently performed causal mediation analysis to determine the proportion of methylation that affects gene expression compared to that of WAA. The Average Causal Mediation Effects score represents the mediation effect of methylation. We also report the 95% C.I., its upper and lower bounds, and the statistical significance of the mediation effect (*p* value).

### Reporting summary

Further information on research design is available in the Nature Research Reporting Summary linked to this article.

## Supplementary information

Supplemental Figures

Supplementary Data 1

Supplementary Data 2

Supplementary Data 3

Reporting Summary Checklist

## Data Availability

The datasets supporting the conclusion of this article are included with the article and in additional files. All raw gene expression data has been submitted to the Gene Expression Omnibus (GEO accession number GSE124076).
