# Supplementary material for: Hepatocyte gene expression and DNA methylation as ancestry-dependent mechanisms in African Americans
Source: NPJ Genom Med. 2019 Nov 25;4:29. doi: 10.1038/s41525-019-0102-y (PMC6877651; doi:10.1038/s41525-019-0102-y)
Supplement: Supplementary file 1 — Supplemental Figures [file 41525_2019_102_MOESM1_ESM.pdf]

Supplemental Figure

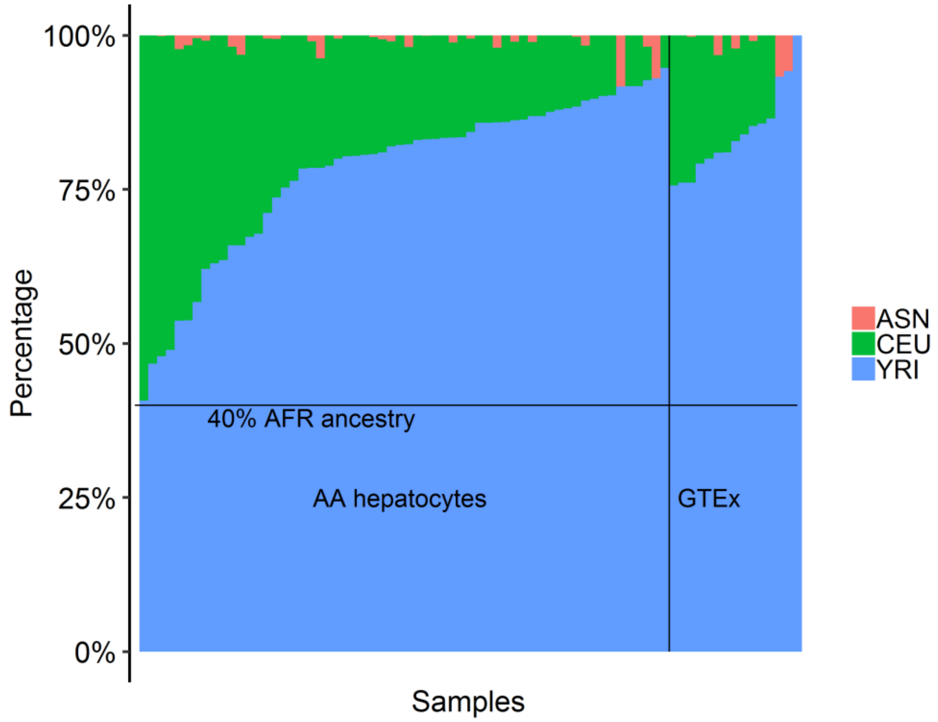

Fig. S1: West African ancestry estimation in the AA Hepatocyte Discovery and GTEx cohorts. An admixture plot showing the ancestry components of the 60 African-American hepatocyte and 15 GTEx cohorts was calculated using fastStructure v1.3. A model with three parental ancestral components (K = 3) was used to estimate from three populations from HapMap phase 3 reference data: Han Chinese in Beijing, China (CHB) and Japanese in Tokyo, Japan (JPT) combined as Asian (ASN, pink); Utah residents with Northern and Western European ancestry (CEU, green); Yoruba in Ibadan, Nigeria (YRI) as African (AFR, blue). Percent WAA is plotted on the y-axis. Each sample is represented individually on the x-axis and partitioned into segments proportional to the contributions of the ancestral components to the individual's genome. The black line identified the threshold use for inclusion in the study.

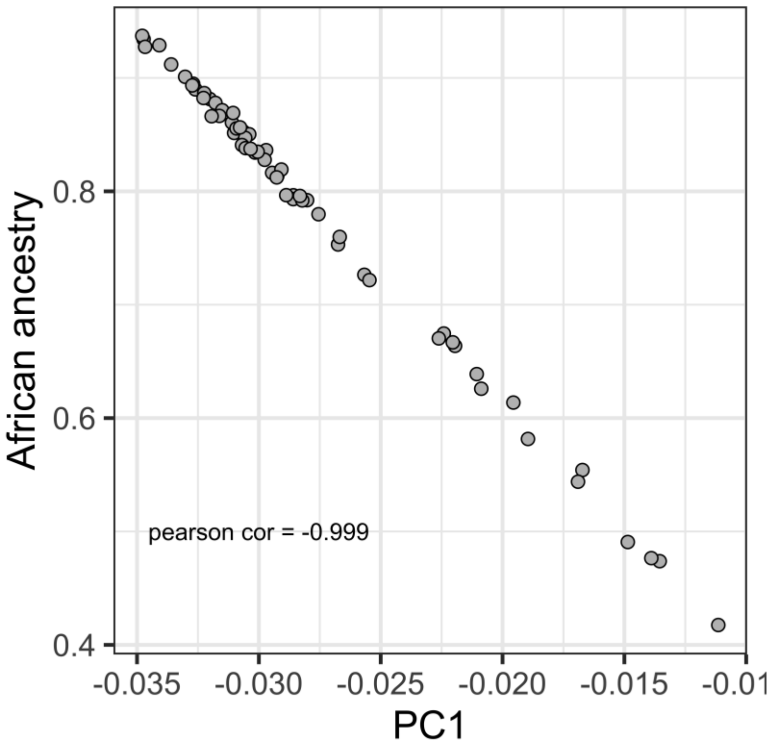

Fig. S2: Correlation plot of proportion of WAA and Principal Component 1 (PC1). A correlation plot of admixture proportion and principal component 1 (PC1) in the 60 African-American hepatocyte samples used in this study (Pearson correlation = -0.999). African ancestry for each sample is represented on the y-axis on a scale of 40% – 100% WAA. Principal component 1 for each sample is represented on the x-axis, in a range between -0.035 and -0.01.

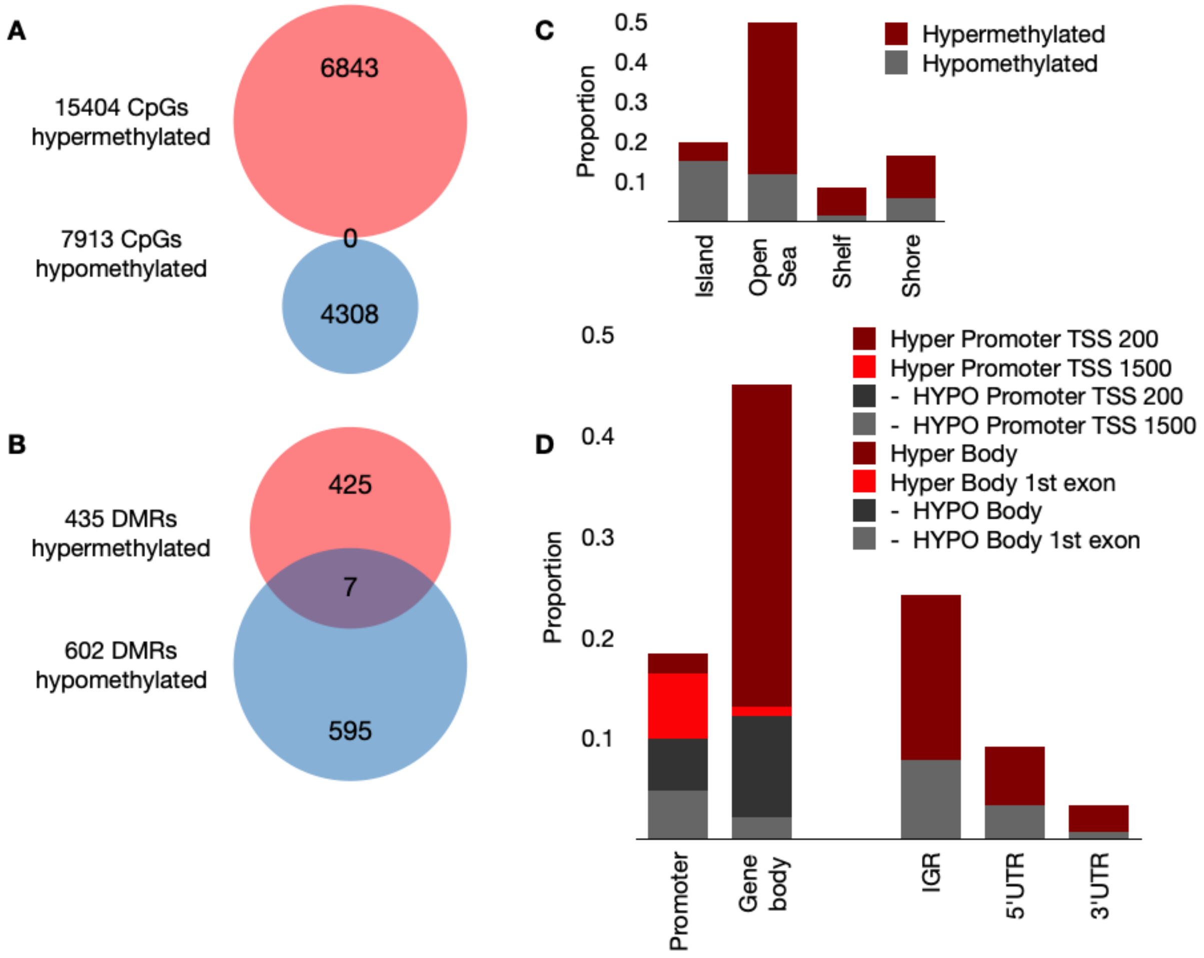

Fig. S3: DNA methylation by genomic features and regions associated with West African ancestry. A. Venn diagrams of unique genes comprised of 15,404 hyper- (pink) and 7913 hypomethylated (blue) CpG sites associated with WAA, and **B.** unique genes comprised of 435 hyper- (pink) and 602 hypomethylated (blue) differentially methylated regions (DMRs) associated with WAA, **C.** Bar graphs showing the proportion of hyper- and hypomethylated CpG sites by genomic features (islands, open sea, shelf and shore), and **D.** proportion of hyper- and hypomethylated CpG sites by transcriptionally-regulated regions: promoter, gene body (with 200 or 1500 bp from the gene transcriptional start site [TSS]), intergenic region (IGR), and 5' and 3' untranslated regions (UTR).

Supplementary Data S1. Genes associated with WAA replicated in Innocenti and GTEx validation cohorts

List of 131 West African Ancestry-associated genes with p-values from the validation of DE gene analysis in the Innocenti Validation Liver Cohort of AAs and EAs and in the AA GTEx Liver cohort.

Supplementary Data S2. GWAS traits and diseases associated with replicated genes

Phenotypic traits associated with replicated genes in the GWAS Catalog.

Supplementary Data S3. WAA-associated genes mediated by methylation

A. Differential methylation analysis results of the ten significant DMRs overlapping the 131 significant WAA-associated genes.
B. Effect size of the ancestry association to gene expression of the five genes containing significant DMRs overlapping the 131 significant WAA-associated genes and with concordant direction of effect.
C. Mediation effects of the five overlapping genes after performing mediation analysis to determine the indirect effect of methylation on gene expression.
